# Elevational Habitat Associations of Tree Species and Deadwood in Ephemeral Streams of the Namib Desert

**DOI:** 10.1002/ece3.74451

**Published:** 2026-09-29

**Authors:** Franziska Messirek, Stephan Getzin

**Affiliations:** ^1^ Department of Ecosystem Modelling University of Göttingen Göttingen Germany; ^2^ Gobabeb Namib Research Institute Walvis Bay Namibia

**Keywords:** Berman test, ephemeral river, *Faidherbia albida*, Kuiseb River, *Moringa ovalifolia*, Tsondab Valley, UAV, *Vachellia erioloba*

## Abstract

In arid and hyper‐arid regions with prolonged dry spells, plants are often particularly dependent on underground water resources. The distribution patterns of riparian tree species along ephemeral streams offer insights into their strategies to optimally utilize water. Here, we monitored the tree species *Faidherbia albida* and *Vachellia erioloba* with drones along the Kuiseb River in the Namib Desert over 6 years, 2020–2025. In addition, *Moringa ovalifolia* trees were monitored in drainage lines in the Tsondab Valley. We retrieved fine‐grained digital terrain models with 1 m resolution to assess the dependency of tree spatial patterns on the lowest lying areas where water runs through. The spatial habitat associations of tree species were assessed with Berman tests. We further analyzed the distribution patterns of deadwood, relative to elevation, for two study plots in the Kuiseb. Our results show that *F. albida* grew in lower lying areas nearer the riverbed, as compared to *V. erioloba* . When both species were highly negatively correlated with elevation in the Kuiseb 1 plot, deadwood amounted to only 0.54%. But when all trees together were positively associated with elevation in the Kuiseb 2 plot, thus growing further up the riverbank, deadwood increased to 12%. Moringa trees in the Tsondab Valley were negatively associated with elevation. They had a preference for low‐lying drainage channels that catch runoff after rainfall. Our research reveals different survival strategies of tree species in streams of the Namib. *Vachellia erioloba* is known for having up to 70 m deep‐reaching roots, allowing the species to grow more independently from riverbeds. *Faidherbia albida* has less deep‐reaching roots, making this species more dependent on riverbeds. Moringas prefer drainage lines but they also benefit from storing water in their thick trunks. Overall, our study highlights that topographical variation and heterogeneity in site conditions may strongly affect tree mortality.

## Graphical abstract

The research presents an in‐depth spatial study of three tree species that grow along ephemeral streams in the arid and hyper‐arid western part of Namibia. The trees form a linear oasis in major streams like the Kuiseb River and provide important shelter and habitat for a variety of plant and animal species.

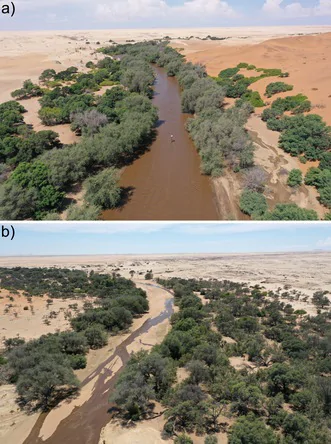

## 1 Introduction

Tree communities along rivers form a vegetation structure and species composition that often differs from the rest of the surrounding area. This characteristic is particularly striking in hyper‐arid regions, where the riparian forest stands out clearly from the surrounding desert landscape. Along ephemeral rivers, it forms a characteristic linear oasis. Here, tree species diversity increases due to the significant rise in water availability. Though, these rivers remain dry for most of the year and carry water for only a few days to weeks during the rainy season in the summer months (Stromberg et al. 2009).

Only well‐adapted tree species can cope with the limited water supply at these sites. They form unique riparian communities that are able to survive under extreme climatic conditions by occupying hydrological niches. Various strategies for water acquisition, such as the development of an adapted root system or long‐term water storage in the trunk, help them to do so. Each species has its own phenological characteristics (Silvertown et al. 2015). Species‐ and site‐specific groundwater use by trees may also reflect hydraulic flexibility because some species increase their groundwater use during drought more than others (Kui et al. 2025).

*Faidherbia albida* (ana tree), *Vachellia erioloba* (camel‐thorn tree), formerly known as *Acacia erioloba* , and *Moringa ovalifolia* (phantom tree) are key tree species in Namibia, adapted to extreme aridity through different ecological strategies. *Faidherbia albida* uses a complex root system to access water in various soil layers (Roupsard et al. 1999) and improves soil fertility through nitrogen fixation (Dupuy and Dreyfus 1992), while its unique phenological cycle efficiently allocates light and water resources. From the Sahelian zone, the tree species is known to be dependent on deep water resources which it can reach with a taproot as deep as 34 m (Dupuy and Dreyfus 1992). In Namibia's ephemeral rivers such as the Hoanib, germination and recruitment of *F. albida* are dependent on flood water (Curtis 2017).

*Vachellia erioloba* is characterized by an exceptionally deep root system that ensures access to groundwater and confers high drought resistance (Mannheimer and Curtis 2009). In the Kalahari, *V. erioloba* attains a rooting depth of up to 70 m, making the survival of this species independent of sporadic rainfall (Fan et al. 2017). Old individuals enrich the soil with nutrients, provide shade, and form important habitats for numerous animal species.

*Moringa ovalifolia* complements this arid vegetation structure through its succulent life form and the ability to store large amounts of water in its trunk. It thus survives even the longest drought periods and plays a decisive role in shaping rocky, nutrient‐poor sites (Olson and Carlquist 2001). Together, these tree species stabilize the ecological balance of ephemeral streams, promote soil fertility, biodiversity, and microclimate formation, and function as keystone species in a hostile environment.

Both historical data and future projections indicate that, due to climate change, Namibia will experience less rainfall, higher temperatures, and longer dry seasons (Atlas of Namibia Team 2022; Attwood et al. 2024). Vegetation dynamics in ephemeral rivers of the Namib may therefore undergo negative changes in the future. In addition, man‐made alterations of the hydrology in catchment areas may also have negative impacts. For example, dieback in *F. albida* in the ephemeral Swakop River north of the Kuiseb is mainly driven by changes in river flow resulting from upstream dam creation (Douglas et al. 2016, 2018). A thorough understanding of hydrological processes, vegetation dynamics, species‐specific characteristics, and resilience to drought is therefore essential to contribute to better forecasting and the protection of these unique ecosystems.

One way to monitor riparian vegetation in rivers like the Kuiseb is the use of satellite images and remote sensing (Normandin et al. 2022). However, high‐resolution drone imagery allows even more precise identification of trees, deadwood, and biodiversity in general (Getzin et al. 2012). For example, dominant woody species like *V. erioloba* , *F. albida* , *Tamarix usneoides*, or *Salvadora persica* have been identified in the Kuiseb with an accuracy of 93%–100% (Morgan et al. 2020). The individual trees with their digitized crowns constitute a point pattern which can be analyzed with spatial statistics to learn about the distribution of a tree species with respect to the surrounding habitat conditions (Punchi‐Manage et al. 2014). In particular, so‐called Berman tests (Berman 1986) are highly suitable to test for spatial independence of a tree pattern from continuous topographic covariates like elevation (Getzin et al. 2014). Such tests are therefore ideal to investigate a tree species' preference to grow either on the lower or the upper riverbanks and to compare different tree species for different study plots. Unlike large‐scale surveys of tree distributions along transects in ephemeral rivers (Douglas et al. 2018), fine‐scale tests of habitat associations of trees allow refined insights into the plot‐level niche requirements of plant species.

So far, tree species of the Kuiseb such as *V. erioloba* and *F. albida* have been only analyzed with regard to their lateral distances from the channel (Morgan et al. 2020). However, analyzing the vertical distance from the deepest part of the river channel is more eligible when interpreting the different rooting depths and growth locations of tree species. This is obvious because the ability to access ground water is inherently a vertical challenge for the plants. Morgan et al. (2020) used a standard digital terrain model (DTM) with 30 m resolution for their analysis. They point out that the line of the lowest elevation of a river course is commonly used in the vectorization of rivers, but due to the lack of accurate DTMs they instead used the geometric midline between the two banks. The midline, however, is not necessarily the deepest point in a riverbed like the Kuiseb because this ephemeral stream can be 50–100 m wide and even more.

To fill this research gap, we focus with our drone survey on an accurate analysis of the growth locations of exemplary tree species, relative to the vertical distribution above the riverbed and above the mean terrain elevation. The aim of this study is thus to analyze the fine‐scale habitat requirements and distribution patterns of major tree species along ephemeral streams in the Namib. *Faidherbia albida* and *V. erioloba* were mapped in two plots of the Kuiseb River (K1 and K2) near the Gobabeb Namib Research Institute, including ground truthing.

In addition, the amount of deadwood was assessed for these two study plots, as well as the change in crown area from 2020 to 2025. The goal was here to provide a detailed comparison of two dissimilar plots with regard to the amount of deadwood, and relate the found differences to the growth locations of trees and to the topographic habitat characteristics. Finally, in the Tsondab Valley, only the distribution pattern of *M. ovalifolia* relative to drainage lines was investigated. Here, the main goal was to study the degree of species dependency on the ephemeral stream, despite having a succulent growth form with a typically thick water‐storing trunk. Specifically, the following three hypotheses were tested:

1.
*Vachellia erioloba* with its exceptionally deep‐reaching roots should be less strongly associated with low elevation areas than *F. albida* because its water supply is more independent from the river channel.
2. The amount of deadwood in the Kuiseb should be larger if trees grow at higher elevation along the river channel because there is less ground water.
3. *Moringa ovalifolia* trees prefer low‐lying drainage lines despite having the ability to store water in their thick trunks.

This approach aims to illustrate the conditional dependence of riparian vegetation on the river course. The resilience of tree species in coping with water deficits determines their adaptability to climate change. It is assumed that the tree species studied differ in their water dependency and thus react to varying degrees to the availability of soil moisture.

## 2 Materials and Methods

### 2.1 Study Sites

The three study sites are located in the Namib Desert of Namibia (Figure 1). The first Kuiseb plot K1 (GPS: 23.565867° S, 15.048796° E) is less than 1 km south‐east of Gobabeb and has a size of 1100 m × 300 m. The second plot K2 (GPS: 23.538006° S, 15.023728° E) is 3 km north‐west of Gobabeb and has a size of 800 m × 400 m (Figure 2). Hence, they have comparable sizes of 33 ha and 32 ha, respectively. The plots are characterized by groundwater‐fed sedimentary soils. Mean annual precipitation (MAP) at Gobabeb is around 25 mm (Eckardt et al. 2013). The plots are thus located in the hyper‐arid Namib. The Kuiseb River originates in the Khomas Hochland Mountains. It has a length of 560 km, a drainage basin of 15,500 km^2^, and it flows only during the rainy season for typically a few days per year (Normandin et al. 2022). During the mapping of trees in the rainy seasons between 2020 and 2025, water was actively flowing only in 2020 and 2022 (Figure 2). The main woody species in the Kuiseb plots are *V. erioloba* , *F. albida* , *T. usneoides* , *S. persica* , and *Euclea pseudebenus* (Morgan et al. 2020). Woody species in plot K1 had a maximal distance of 150 m to the riverbed, while in plot K2 this distance was more than double.

**FIGURE 1:**
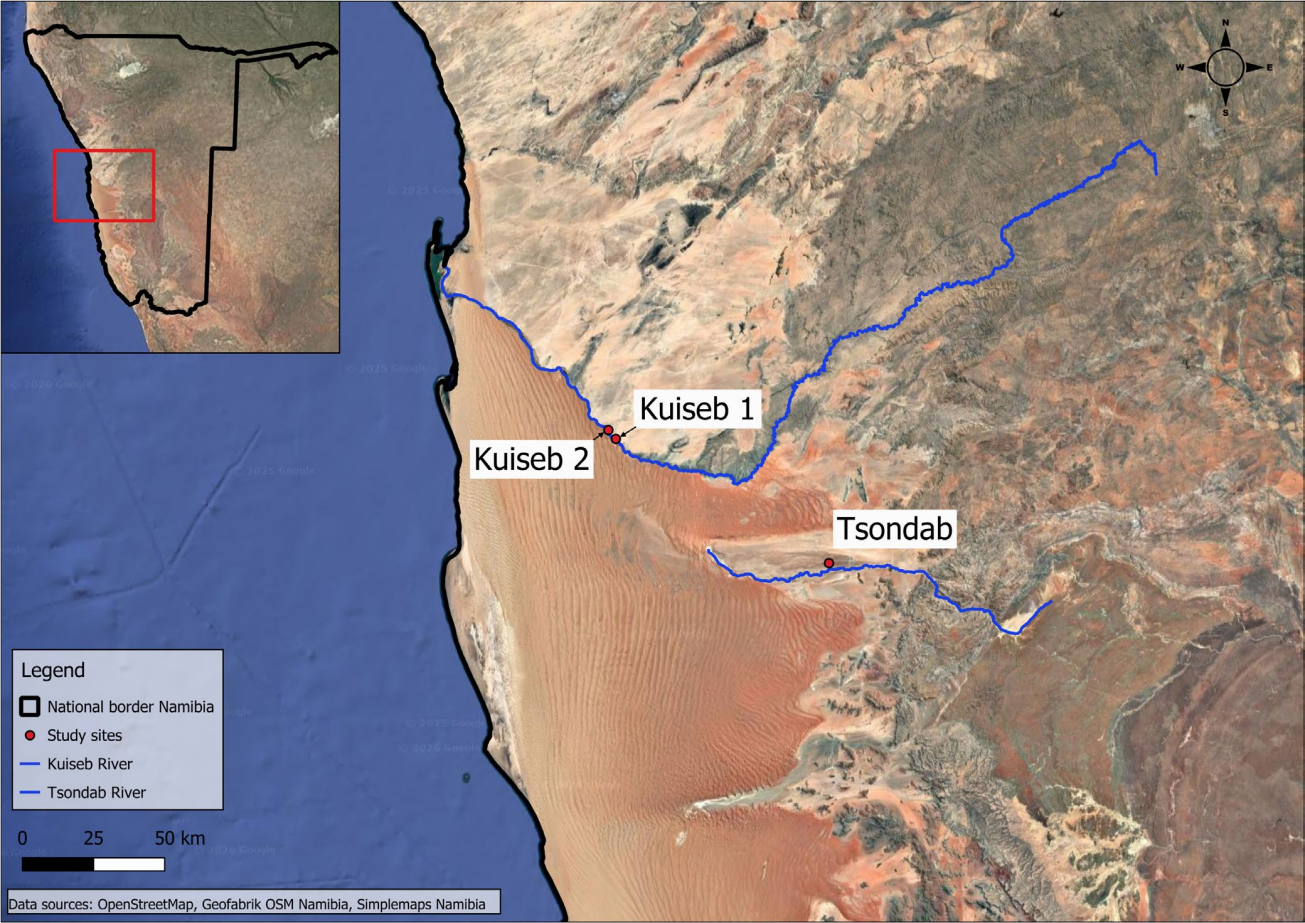
Map with the study sites in the ephemeral Kuiseb River and near the Tsondab River.

**FIGURE 2:**
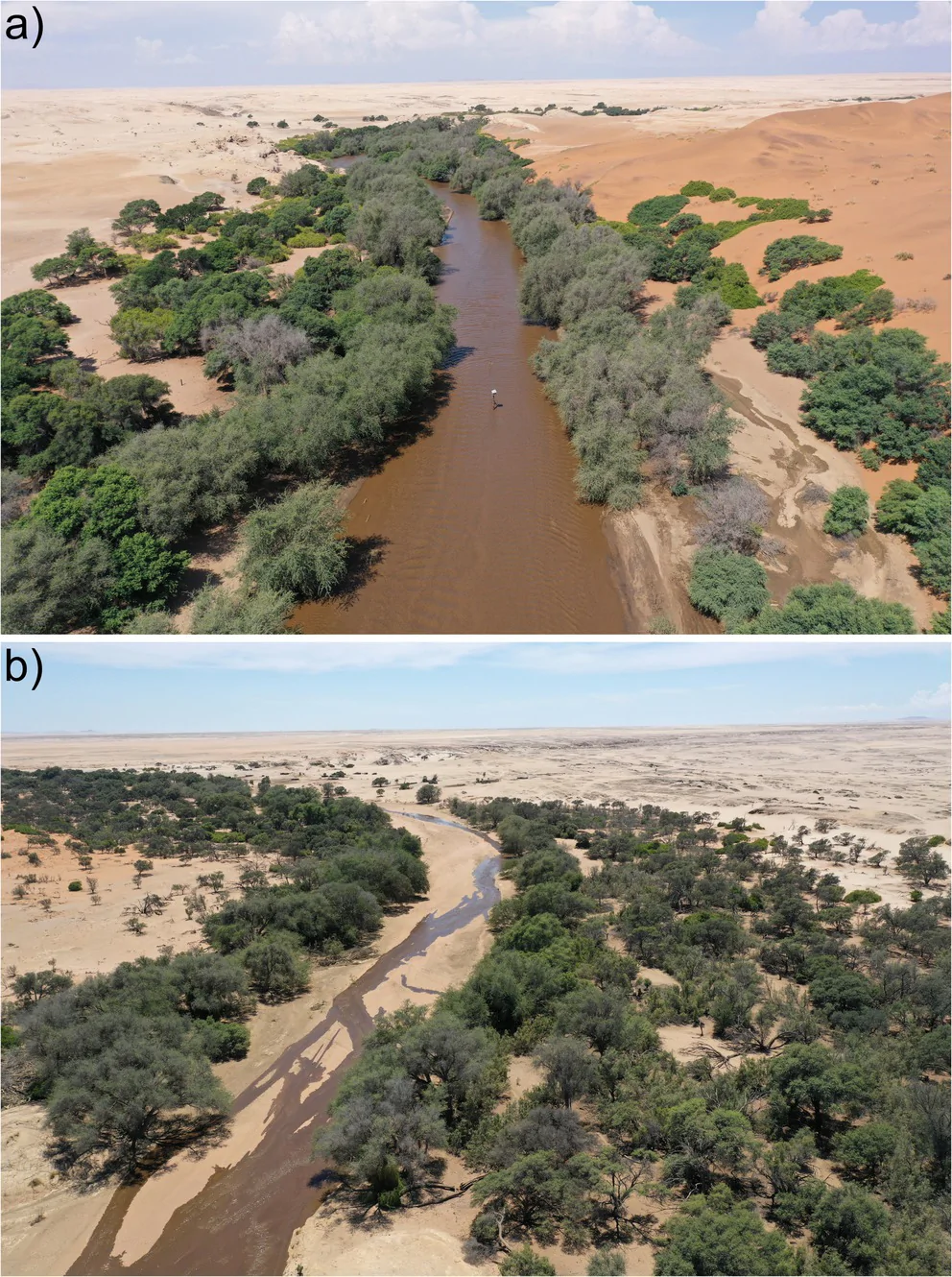
The Kuiseb 1 plot in full flood on 19 February 2022 (a). Light gray, open tree crowns growing next to the river are *F. albida* . Bright green, 2–4 m low shrubs on the sandy dunes are *S. persica* . Dark green, dense crowns are predominantly *V. erioloba* . The Kuiseb 2 plot with some water in the lowest part of the riverbed on 27 February 2020 (b). Note that the midline between the two banks is not the deepest part of the river. Compared to the plot K1, woody vegetation in K2 grew with more than 300 m distance to the riverbed more than twice as far away (all photos taken by Stephan Getzin).

The Tsondab plot (GPS: 23.958496° S, 15.785839° E) with the *M. ovalifolia* trees is on farm Vito, 23 km south‐west of Solitaire and 2 km north of the ephemeral Tsondab River. MAP in that area of the Tsondab Valley is about 80 mm and the soils on sandstone substrate have a hard calcareous layer (Getzin et al. 2024). The plot has a size of 200 m × 200 m and is traversed by a 10–20 m wide drainage channel that runs parallel to the Tsondab River from east to west (Figure 3). The study site is almost exclusively dominated by single‐standing moringa trees.

**FIGURE 3:**
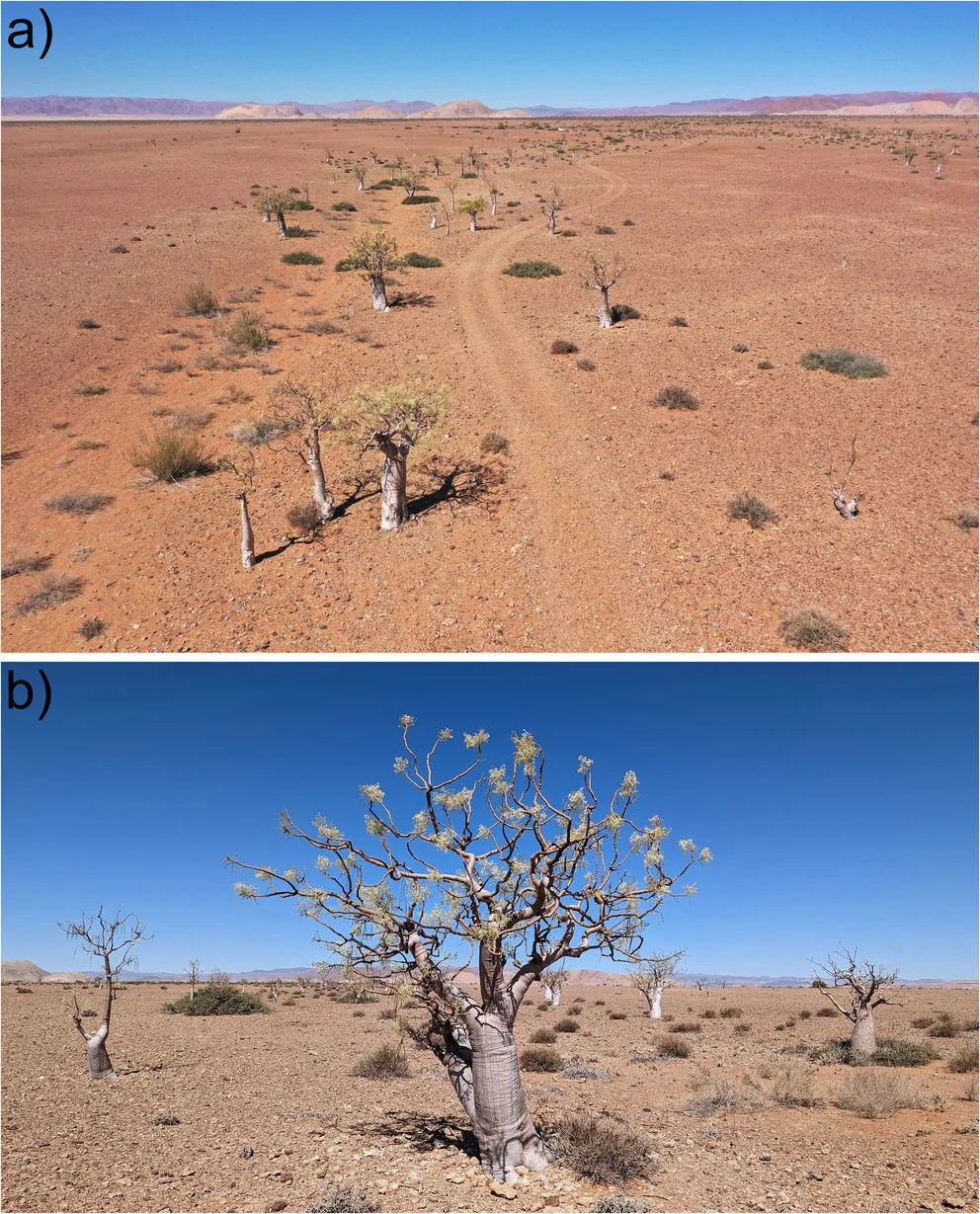
View over the Tsondab plot with moringa trees growing along drainage channels (a). *Moringa ovalifolia* trees can have trunks up to 1 m in diameter, in which they store water (b).

### 2.2 Data Collection

High‐resolution aerial images were captured for all three study areas using a drone (DJI Mavic 2 Pro) with flights conducted at an altitude of 90 m over areas K1 and K2 on the Kuiseb River and at 45 m over the Tsondab plot with moringa trees. This resulted in orthorectified images with resolutions of 3 cm and 2 cm per pixel, respectively. The ortho‐images were produced with OneButton (Standard Edition 5.2, www.icaros.us) software. The K1 plot was repeatedly mapped over six consecutive years 2020–2025 during the rainy season from February to April (Figure 4). The other two plots were less often mapped. The drone‐based segmentation of tree crowns and of deadwood used in this study is based on the year 2024 (K1: 19 February 2024, K2: 12 March 2024, Tsondab: 17 March 2024).

**FIGURE 4:**
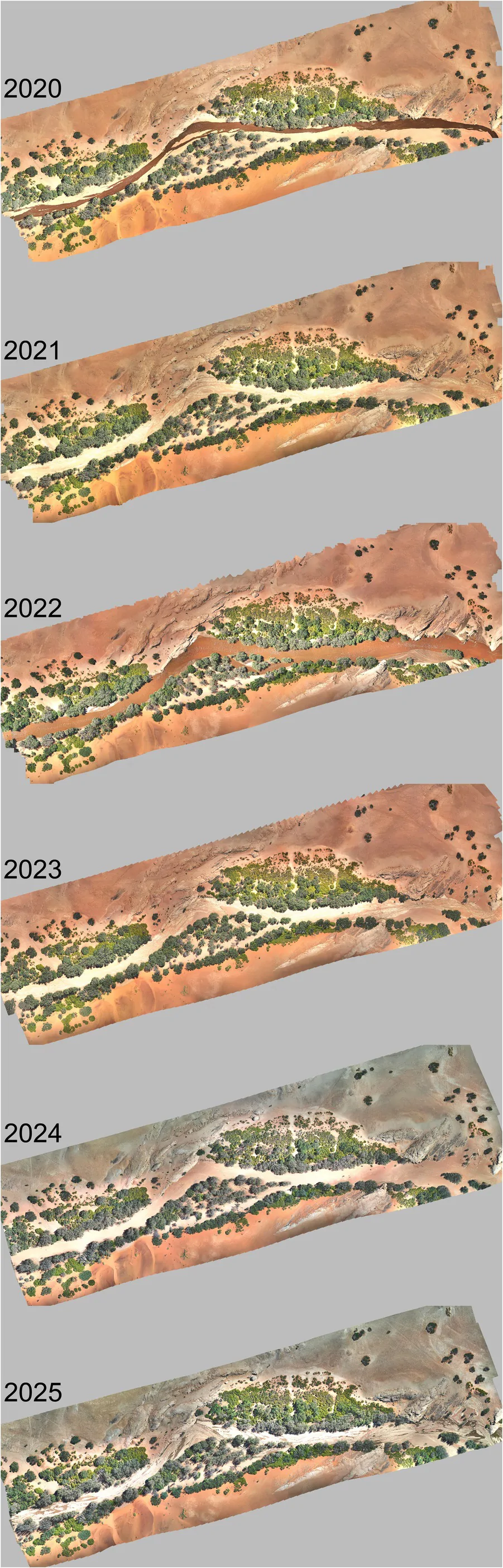
Repeated drone‐mapping of the Kuiseb 1 plot near Gobabeb. The river was flowing during fieldwork in 2020 and 2022. The weak flow in 2020 indicates the deepest section of the river channel and thus the low‐lying habitat with most easily accessible water.

In the two Kuiseb plots, more than 100 individual trees were identified on the ground with species names and their positions were recorded via GPS to validate the aerial image interpretation. This included the main tree species *V. erioloba* , *F. albida* , *T. usneoides* , and *E. pseudebenus*. Likewise, in the Tsondab plot, more than 60 individual *M. ovalifolia* were mapped in the field for ground‐truthing.

We only segmented the tree crowns of the two largest and most dominant species in the Kuiseb, *V. erioloba* and *F. albida* , which are characterized by dark green, dense crowns and light gray, open canopy, respectively (Appendix: Figure A1a,b, Figure A2a,b). Drone images from other years with different light conditions were also used to help in identifying individual trees. The segmentation of individual trees may, however, contain a certain error, since the canopies appear partly overlapping in the images (Morgan et al. 2020). This potential error is negligible for our study, since we are not interested in testing the spatial interaction between individual trees, that is the meter‐scales of potential facilitation or neighborhood competition, leading to opposing patterns such as clustering or regularity, respectively (Getzin et al. 2006).

But here, we test the overall habitat association of a species with its average distribution in the study plot. Consequently, a potential error in mapping, for example, only one individual even if there were two, is negligible since this error would occur evenly across all trees and elevations within the plots. Individual trees were more easily segmented in the Tsondab plot, as the moringa trees are more solitary and the shade of the crown's branches is well visible in the drone images (Appendix: Figure A2c).

Tree crowns were manually delineated with QGIS 3.36 software (https://qgis.org) for all three study plots to obtain shapefiles in a vector format, containing geo‐referenced information on the x, y‐coordinates of the crown centers and on the crown area (Figure 5a). Likewise, all visible deadwood was segmented for the two Kuiseb plots based on the year 2024, as this was the reference year for ground‐truthing and species identifications in drone images. Similarly to the tree crowns, deadwood was segmented as polygons. Where larger amounts of deadwood accumulated on the ground, it was digitized as one polygon. An example for typical occurrences of deadwood is shown in Figure 6 and Appendix: Figure A1c, Figure A2a,b. Note that deadwood in this context refers primarily to woody stems lying flat on the ground. Other forms of mortality, such as standing dead trees, may be underdetected in this aerial‐image survey.

**FIGURE 5:**
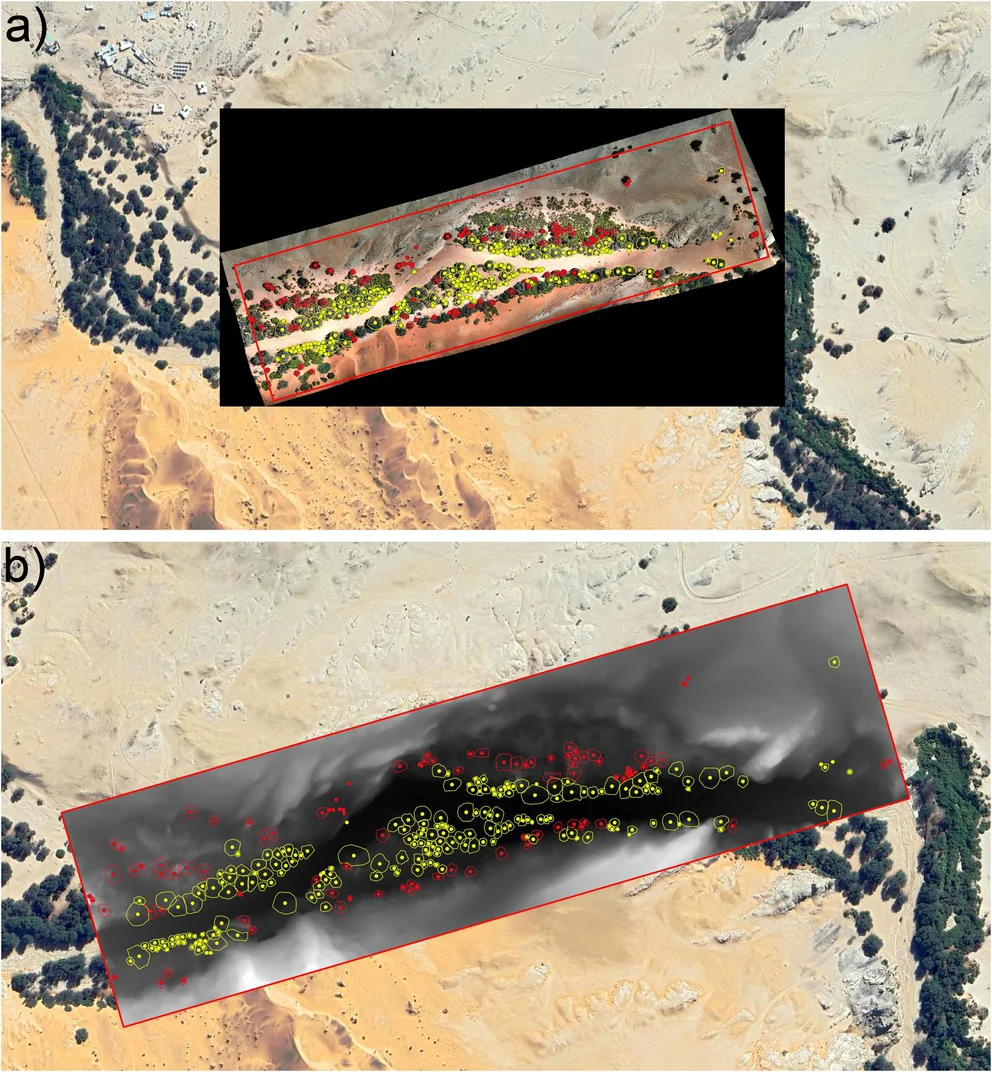
The Kuiseb 1 plot, next to the Gobabeb Namib Research Institute, with delineated tree crowns and crown centroids of *Vachellia erioloba* (red) and *Faidherbia albida* (yellow). The red line shows the borders of the 1100 m × 300 m plot. The drone image from 2024 has a resolution of 3 cm/pixel (a). The digital terrain model (DTM) has a resolution of 1 m/pixel and dark color shows the deepest habitat sections (b).

**FIGURE 6:**
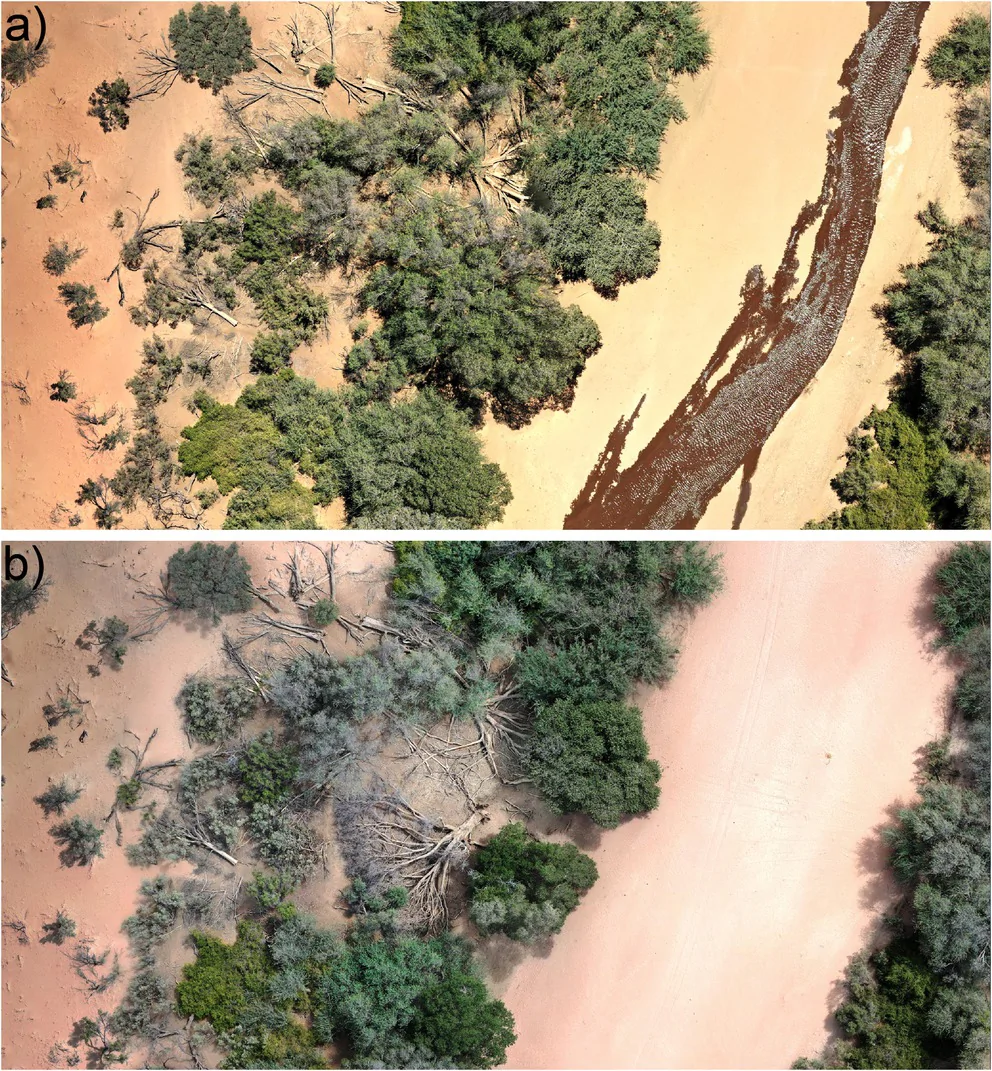
Example of tree mortality in the Kuiseb 2 plot. In 2020, the *F. albida* tree in the center of the image was still alive (a) but in the 2024 census it appeared as deadwood (b).

In addition, we were interested in potential changes of crown areas of trees in the Kuiseb. For this reason, tree crowns were digitized on a selective basis. This was a species‐independent digitization in which individual tree species were not taken into account. Therefore, this dataset allows only area‐based conclusions regarding biomass growth to be drawn. Tree crowns in the plots K1 and K2 were digitized from the first aerial photographs taken on 26 February 2020, and the same specimens were re‐recorded in the aerial photographs from 12 April 2025. The sample size comprised 50 tree crowns per plot. The selection of trees consisted of free‐standing, easily recognizable tree crowns distributed across the entire study plot, enabling reliable delineation and analysis of individual crown areas. Subsequently, the difference in crown area between the two years 2020 and 2025 was calculated to determine the area‐based growth.

To assess habitat associations of trees, a digital terrain model (DTM) was created for each plot, based on the drone‐mapped images and processing in OneButton software. For this purpose, the trees along the riverbeds had to be cut out as polygons because they confounded the true elevation of the terrain. Subsequently, the holes were filled with surrounding ground‐elevation data via interpolation. This was done with the tool “Fill nodata” in GDAL (Geospatial Data Abstraction Library). Finally, fine‐grained DTMs with 1 m resolution were created that represented well the river channel and the surrounding topography (Figure 5b).

### 2.3 Data Analysis

The spatial patterns of *V. erioloba* , *F. albida* , and *M. ovalifolia* , of *V. erioloba* and *F. albida* combined, and of deadwood in the Kuiseb plots were analyzed with Berman tests (Berman 1986) to determine the habitat associations with regard to elevation and thus river topography.

In this test, the observed distribution of the values *S*_*obs*_ of a spatial covariate *Z* at the tree data points *x* and the predicted distribution of the same covariate values *S*_sim_, generated by a null model of complete spatial randomness (CSR), are compared using the *Z* test statistic. Randomization with the test is done for the entire observation window, and results are robust, irrespective of the size of the plots. In the conventional Berman test, *Z*_1_ is computed based on the mean *S*_obs_ of the covariate values at all data points *x*: *Z*_1_ = (*S*_obs_ − *μ*)/*σ* where *μ* is the mean value of *S*_sim_ under the null model and *σ* ^*2*^ the corresponding variance (Getzin et al. 2014). The null distribution of this test statistic is approximately the standard normal distribution.

However, the *Z*_1_ statistic is a comparison between the two mean values, and is not sensitive to the type of deviation, such as the symmetry, between two distribution curves (Baddeley et al. 2016). Therefore, the *Z*_2_ variant of the Berman test can produce more insightful results. The test statistic Z_2_ is computed as follows: The values of the covariate at all locations in the observation window, weighted by the point process intensity, are compiled into a cumulative distribution function *F*. The probability integral transformation is then applied: The values of the covariate at the original data points are transformed by the predicted cumulative distribution function *F* into numbers between 0 and 1. If the model is correct, these numbers are uniform random numbers and the statistic *Z*_2_ is the standardized sample mean of these numbers (Baddeley et al. 2024). Values of *Z*_2_ between −1.96 and 1.96 correspond to a *p*‐value > 0.05 and encircle cases without significant departures of the null model. In contrast, lower or higher values of the *Z*_2_‐statistic show significant negative or positive associations with the covariate (elevation), respectively. All analyses were done using the software *R* (package *spatstat*; http://www.R‐project.org/).

## 3 Results

### 3.1 Elevational Habitat Associations of Tree Species in the Kuiseb Plots

The measured elevations vary across the study sites: In K1, they range from 397 to 430 m above sea level, with a mean of 406 m, while K2 ranges from 388 to 407 m, with a mean of 392 m (Table 1, Appendix: Figure A3). *Vachellia erioloba* , *F. albida* , and both species combined were all significantly negatively correlated with elevation, and their mean elevations were below the mean height of 406 m of the plot. With 401 m, *F. albida* grew on average 5 m below the mean elevation of the terrain, corresponding with a strongly negative *Z*_2_‐value of −14.889 (Table 1). *Vachellia erioloba* grew on average 3 m higher than *F. albida* in the river valley and had a weaker negative *Z*_2_‐value of −5.0989.

**TABLE 1:** Results of Berman tests and habitat properties. Significant negative and positive *Z*_2_‐values indicate negative and positive associations with elevation, respectively. Mean elevation at *Z*x is the average terrain height, where tree‐species positions and deadwood have been recorded in the plot. The mean, min, and max elevation values of *Z* show the topographical terrain height of the plots above sea level, as obtained from the drone‐based DTM.

Study site	Tree species	Number of individuals	*Z*_2_	*p*	Mean elevation *Z*x (m)	Mean elevation *Z* (m)	Min elevation *Z* (m)	Max elevation *Z* (m)
**Kuiseb 1**	*F. albida*	213	−14.889	< 0.001	401	406	397	430
	*V. erioloba*	102	−5.0989	< 0.001	404			
	All trees	315	−15.252	< 0.001	402			
	Deadwood	54	−4.7391	< 0.001	403			
**Kuiseb 2**	*F. albida*	61	−1.9572	0.05032	392	392	388	407
	*V. erioloba*	151	4.9673	< 0.001	393			
	All trees	212	3.14	0.00169	392			
	Deadwood	489	8.2196	< 0.001	393			
**Tsondab**	*M. ovalifolia*	116	−3.2711	0.00107	874	874	873	876

The habitat associations of trees differed considerably in the Kuiseb 2 plot. There was no significant negative correlation of tree species with elevation. While *F. albida* had again a negative *Z*_2_‐score of −1.9572, it was just not significant (*p* = 0.05032) and the species had a mean elevation value of 392 m being equal to the mean terrain height (Table 1). *Vachellia erioloba* was significantly positively associated with elevation, growing above the mean terrain height.

### 3.2 Elevational Habitat Associations of Deadwood in the Kuiseb Plots

Deadwood in the Kuiseb 1 plot was significantly negatively correlated with elevation and its mean elevation was 3 m below the mean height of the plot. In contrast, deadwood in K2 had a highly significant, positive *Z*_2_‐score of 8.2196, indicating that most dying trees were found above the mean terrain height at an elevation of 393 m.

Only 54 localities of deadwood were found in the Kuiseb 1 plot and that occurred also at lower‐lying areas 3 m below the mean terrain height. The area‐wise cover of deadwood amounted to only 0.54% of the 33‐ha plot, while both tree species together covered 12.16%.

Remarkably, also the amount of deadwood differed extremely in both plots: In K2, 489 localities of deadwood were found and their area‐wise cover made up 12.08% in this 32‐ha plot (Table 1). Hence, the amount of deadwood had a percentual cover that was more than 20 times higher than in K1 (Appendix: Figure A2a,b). By contrast, the total coverage of the two segmented tree species was with 11.39% almost the same as the 12.16% in the K1 plot.

The additional assessment of changing crown areas from 2020 to 2025 revealed also some differences between the two Kuiseb plots, but the 50 sampled trees increased in both study sites their crown areas, despite the large differences in deadwood. In K1, the crown areas enlarged from a mean value of 86.6 sqm in 2020 to 103.7 sqm in 2025, with a total area‐wise increase of 851 sqm (Appendix: Table A1). Of the 50 trees, six showed a negative increase in crown area. In K2, the crown areas enlarged from a mean value of 87.8 sqm in 2020 to 114.9 sqm in 2025, with a total area‐wise increase of 1355 sqm. Of the 50 trees, only three showed a negative increase in crown area. Thus, the increase in crown area was about 1.6 times larger in K2, as compared to K1.

### 3.3 Elevational Habitat Association of Moringa Trees in the Tsondab Plot

At the small Tsondab plot, elevations range only from 873 to 876 m, with a mean of 874 m (Table 1, Appendix, Figure A3). The terrain has thus more characteristics of drainage lines rather than true riverbeds. Nevertheless, the 116 digitized *M. ovalifolia* showed a negative association with terrain elevation because their *Z*_2_‐value was −3.2711 significantly negative.

## 4 Discussion

This study on the spatial patterns and habitat associations of tree species investigates the niche requirements of trees with regard to water availability in temporarily flooded riverbeds. The three study plots differ in that the Kuiseb sites are true ephemeral rivers while the Tsondab plot, with its low elevational differences, reflects rather drainage lines. Detailed point pattern statistics of plant distributions are ideal to learn about different demographics in these trees growing along ephemeral streams of the Namib Desert. The in‐depth comparison of many tree individuals in exemplary study plots allows thereby generalizable insights into the effects of heterogeneities on tree survival and demographics (Getzin et al. 2008).

### 4.1 Elevational Habitat Associations of Tree Species in the Kuiseb Plots

We hypothesized that *V. erioloba* with its exceptionally deep‐reaching roots should be less strongly associated with low elevation areas than *F. albida* because its water supply is more independent from the river channel. This hypothesis was confirmed by the study. Along the Kuiseb River, it is evident that *F. albida* , but to some extent also *V. erioloba* , occurs primarily in deeper sections of the river valley, where soil moisture is highest due to groundwater and seasonal flooding (Morgan et al. 2020). *Faidherbia albida* is strongly tied to the deeper riverbed due to its more lateral root system near the surface and the less deep taproot (Dupuy and Dreyfus 1992), making the species dependent on regular access to groundwater. In contrast, *V. erioloba* can also grow in higher, drier areas, as its deep taproot allows it to access water even when it is scarce. The species is known to have exceptionally deep roots (Fan et al. 2017), which is also the reason why *V. erioloba* can be sometimes found in the most isolated places of the Namib Desert. These different hydrological niches allow both species to coexist in the same area, with *F. albida* primarily colonizing the active river zone and *V. erioloba* tolerating higher elevations.

The different hydrological niches are also affecting the long‐term survival of these species and their vulnerability to negative changes in water management. For example, dieback in the ana tree *F. albida* was in the ephemeral Swakop River most severe where trees grew on drier stretches of the river, where tributary flow was absent (Douglas et al. 2016). In that large‐scale study less than 100 km north of the Kuiseb, dieback in *F. albida* was primarily driven by changes in river flow resulting from upstream dam creation.

While the study of Douglas et al. (2016) compared tree mortality at 24 sites in 20 m wide transects, covering more than 250 km of the river's length, our research on tree mortality focuses more on the detailed differences in growth locations and topography. This has the advantage that population dynamics, biomass growth and tree mortality can be directly related to the fine‐scale characteristics of the riparian habitat. Our DTM with 1 m resolution allows refined insights into the different constraints that trees are exposed to in different—even nearby—sections of the Kuiseb. This is also visually shown in our Figure 2b, which indicates that when water levels are very low, the river flows on only one side of the channel. Both fine‐scale topographic characteristics of the channel and of the riverbanks may be important for the population dynamics of trees and may explain why some inventory plots have a high tree mortality while other nearby plots have a low mortality. This leads us to our second hypothesis.

### 4.2 Elevational Habitat Associations of Deadwood in the Kuiseb Plots

We hypothesized that the amount of deadwood in the Kuiseb should be larger if trees grow at higher elevation further up from the river channel. We investigated this hypothesis by analyzing two very comparable 33 ha and 32 ha plots that are only separated by a distance of 4 km. These Kuiseb plots have the same climate, tree species composition and they receive the same amount of water, once flooding occurs during the rainy season. They are also both part of the Namib‐Naukluft Park and thus protected by the same conservation status. In addition, also the crown cover of the segmented species *V. erioloba* and *F. albida* was with 12% in the K1 plot and 11% in the K2 plot nearly identical. However, the percentage of deadwood was with 12% coverage in the K2 plot more than 20 times higher than the 0.5% in the K1 plot. This highly dissimilar result cannot come from large‐scale differences in water management upstream but it is primarily due to heterogeneity in local topographic site conditions.

The very low amount of deadwood in K1 had a mean elevation that was 3 m below the mean of the terrain (Table 1). In contrast, the high amount of deadwood in K2 was found at a mean elevation that was 1 m above the mean of the terrain. In addition, while the *Z*_2_‐value of the Berman test was negative for *F. albida* , indicating a preference for lower lying areas, it was not significant in K2. Thus, trees in K2 were forced to either grow at the same height as the mean terrain elevation or, in the case of *V. erioloba* , even above. The highly significant *Z*_2_‐value of 8.2 for deadwood in K2 indicates that tree mortality can be mainly attributed to growing too far above the accessible groundwater. Especially during prolonged years of drought and insufficient flooding, trees at those higher elevations cannot survive. For example, the Kuiseb around Gobabeb had no flow in the summers of 2007, 2010, 2013, 2016, and 2019 (Morgan et al. 2021). Since our segmented deadwood consists of fallen trees and shrubs that lie flat on the ground and have no chance of recovery, the results regarding tree mortality are particularly striking.

We do not know the reasons why trees grow on higher elevations in K2. Germination and recruitment depend on flood water in arid regions but “floods are both a blessing and a curse to the riparian vegetation” because heavy discharges may uproot mature trees and flatten or drown younger trees (Curtis 2017). It is also possible that the river changed its course over years and decades, resulting in an increased separation of the trees at the elevated plateau from the active channel (Figure 2b). However, this is merely speculation.

The results have implications for the selection of representative study sites in comparative landscape‐scale analyses across regional gradients and for the spatial extrapolation of tree mortality, biomass, or carbon fluxes. For example, biases in field studies of forest structure may be strong in tropical America or Africa, resulting in a considerable sampling error of the small field plot to approximate the forest variable in the surrounding landscape (Marvin et al. 2014; Getzin et al. 2017). Overall, our study demonstrates that mortality rates of riparian trees can be highly dissimilar in even close‐by inventory plots due to differences in topographical habitat conditions. In that, riparian forests are very similar to other types of, for example, temperate forest on Vancouver Island, where successional dynamics are intensified in heterogeneous forest stands with strong spatial structures, resulting in cascading feedback effects of plant demographics, interacting with spatial structure (Getzin et al. 2008).

Even though we did not study the effects of heterogeneity like differences in soil texture or microclimate, the intensified successional dynamics appear also evident from the increase in crown areas in K2. Irrespective of the high mortality, the increase in crown area from 2020 to 2025 was even stronger in that plot than in K1. The 50 sampled trees enlarged in K2 by 1355 sqm, while in K1 it was only 851 sqm. These results are similar to the above‐mentioned study of temperate forests where adult growth was faster but adult mortality was also higher. Getzin et al. (2008) hypothesized that the dynamics of their studied heterogeneous forest stand were faster due to asymmetric competition with disproportionate benefit to taller plants.

Possibly, the higher mortality in K2 can also be a result of increased competitive pressure among neighboring trees. Even though the higher proportion of deadwood in K2 initially suggests poorer site conditions, this can have a positive effect on the living vegetation. In studies conducted in a semi‐arid river system in Kruger National Park, Pettit and Naiman (2006) found that deadwood had a positive effect, particularly for young trees, as it acts like a sponge and stores water and nutrients even during the long dry season. It promotes the formation of ecological niches that support the regeneration of the forest ecosystem and are part of natural forest development. Overall, the increase in crown areas over the years agrees with previous studies. For the Kuiseb River around Gobabeb, Morgan et al. (2021) found a 33% increase in vegetation cover between 1984 and 2019, based on analyzing Landsat satellite images.

### 4.3 Elevational Habitat Association of Moringa Trees in the Tsondab Plot

In our third hypothesis we suspected that *M. ovalifolia* trees prefer low‐lying drainage lines despite having the ability to store water in their thick trunks. Very little is known about the population dynamics of the species *M. ovalifolia* . It is the only moringa species that grows naturally in Namibia but quantitative data analyses on its distribution are lacking. A related species from India, *M. oleifera* , is a fast‐growing drought‐resistant tree that is artificially grown as fodder trees in orchards in Namibia (Korsor et al. 2019). *Moringa ovalifolia* grows typically in the more arid and semi‐arid western regions of Namibia where it is found on rocky mountain slopes. Occasionally it is also found on plains such as in the Etosha National Park where it even forms a small forest, known as the “Phantom Forest” or “Sprokieswoud” (Mannheimer and Curtis 2009). Our study site in the Tsondab Valley is another rare example from Namibia, where *M. ovalifolia* forms a small forest on plains.

Our hypothesis about its preference for low‐lying areas was supported by the statistical analysis because the moringa trees had a significantly negative *Z*_2_‐score of −3.3. Unlike for the Kuiseb, the low elevational range at the Tsondab plot reflects drainage lines rather than true riverbeds. Nevertheless, small elevational differences are important for the ecohydrology, as drainage lines accumulate disproportionally more runoff water during rainfall events. This benefits also *M. ovalifolia* , even though the species is well‐adapted to drought. In contrast to the drought‐resistant *M. oleifera* , the native moringa species *M. ovalifolia* grows slower in height, probably because it invests more biomass into underground growth of roots to preserve energy during drought conditions (Korsor et al. 2019). Together with its high water‐storage capacity in the trunk and roots, this gives moringa a certain degree of independence from rainfall (Olson and Carlquist 2001). Nevertheless, the preference of *M. ovalifolia* for drainage lines reveals that such significant habitat associations are adaptations of the whole population to potential drought, similar to the riparian tree species in ephemeral river systems as the Kuiseb.

## 5 Conclusions

The groundwater reservoirs beneath ephemeral streams such as the Kuiseb and the Tsondab are primarily responsible for supplying tree species with sufficient water in deserts. *Faidherbia albida* and *V. erioloba* depend on these water resources and form green linear oases. Each tree species has developed its own species‐specific strategy to adapt to the dry climate and arid sites. *Moringa ovalifolia* benefits from drainage lines near the Tsondab riverbed but is not strictly dependent on it, as it is quite capable of storing large amounts of water in its trunk over an extended period. *Faidherbia albida* is heavily dependent on regular flooding along the Kuiseb and is therefore particularly sensitive to a lack of rainfall or negative changes in the water management upstream. Its occurrence along the riverbank is mainly in low‐lying areas that offer high water inflow and good access to groundwater. In contrast, *V. erioloba* is a generalist species with a greater capacity to adapt to environmental changes, as evidenced by its wide distribution. It requires the river course but also spreads into higher elevations due to its particularly long roots.

Our study highlights that the amount of deadwood in ephemeral rivers like the Kuiseb can be strongly dependent on individual site conditions like the topography. Our nearby replicate plots in the Kuiseb, which share the same climate, differed markedly in the dieback of trees, which we mainly attribute to local differences in favorable or unfavorable survival templates. These habitat differences may be induced by a change in the river course over long or even short time spans. They may also reflect natural heterogeneity in the landscape. Thus, if the dieback of trees needs to be assessed, a careful selection of representative study plots is required.

## Author Contributions

**Franziska Messirek:** data curation (equal), formal analysis (equal), investigation (equal), writing – original draft (equal). **Stephan Getzin:** conceptualization (lead), data curation (equal), formal analysis (lead), investigation (equal), methodology (lead), project administration (lead), writing – original draft (supporting), writing – review and editing (lead).

## Funding

We are grateful to the German Research Foundation (DFG), Deutsche Forschungsgemeinschaft for supporting this research (433057155 and 508387707).

## Conflicts of Interest

The authors declare no conflicts of interest.

## Figures and Tables

**FIGURE A1:**
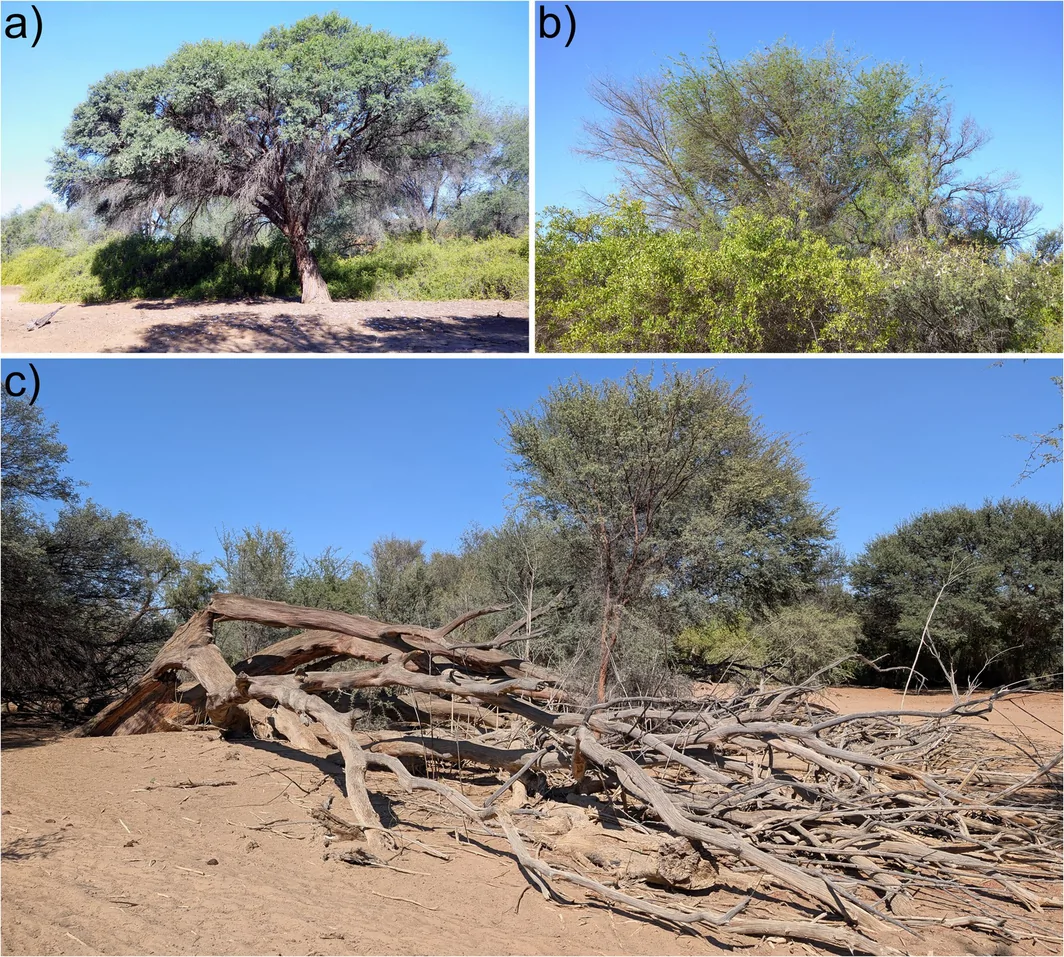
Example of *Vachellia erioloba* in the Kuiseb 1 plot (a). Note the relatively dense crown and the bright green shrub *Salvadora persica* below. Example of *Faidherbia albida* in the Kuiseb 1 plot, which has a more open crown (b). Example of deadwood in the Kuiseb 2 plot (c).

**FIGURE A2:**
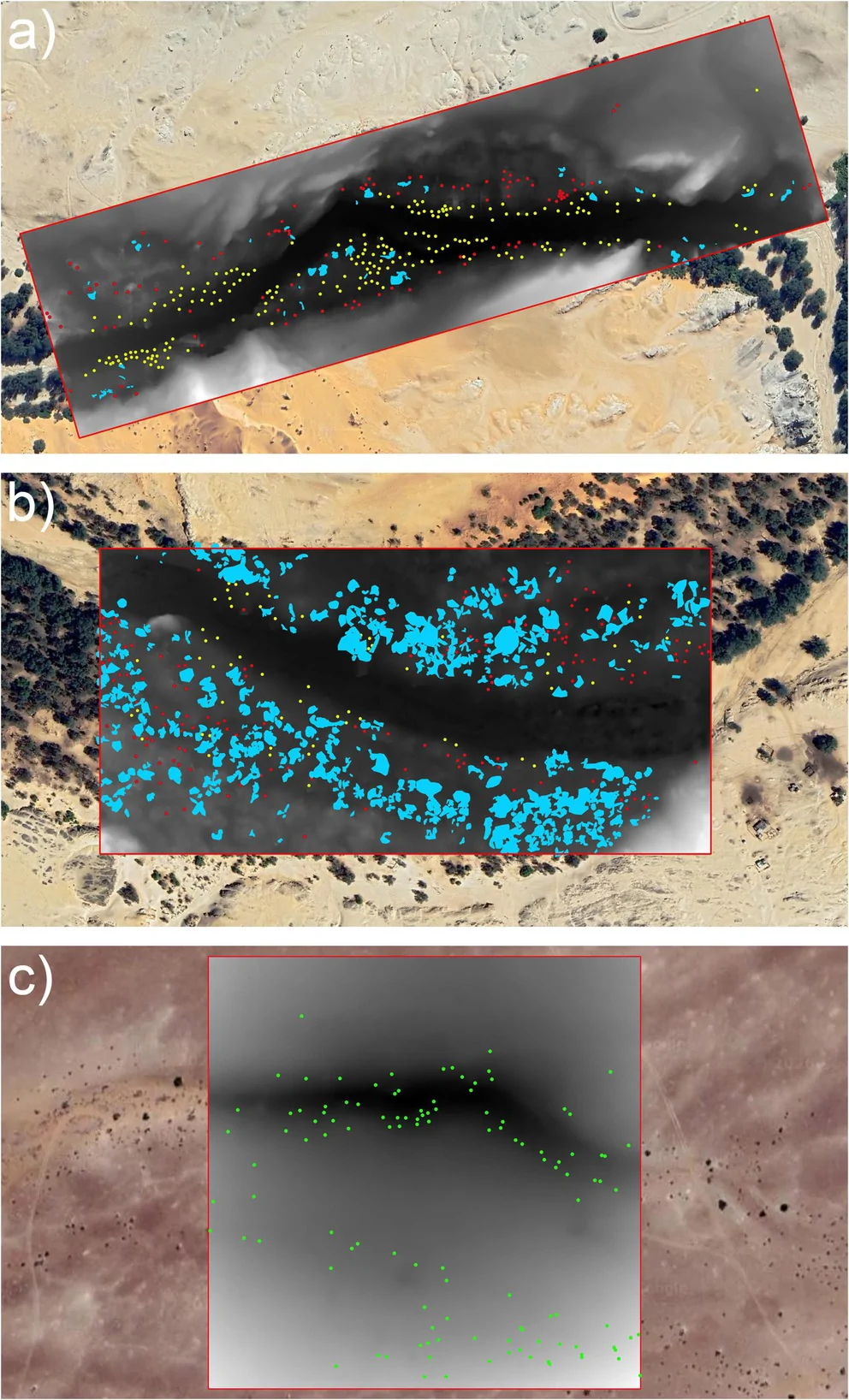
Digital terrain model with digitized crown centroids of *Vachellia erioloba* (red), *Faidherbia albida* (yellow) and with polygons of delineated deadwood (blue) in the Kuiseb 1 plot (a). Same description as for the Kuiseb 2 plot (b). Digitized *Moringa ovalifolia* (green) and the DTM are shown for the Tsondab plot in (c).

**FIGURE A3:**
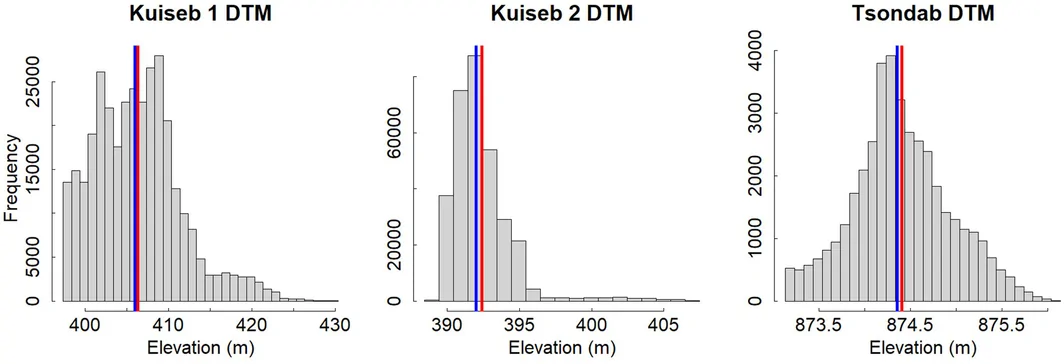
Histograms of drone‐derived digital terrain models (DTMs) with 1 m resolution for the three study plots. Mean and median elevation values are shown with red and blue lines, respectively.

## Data Availability

The drone‐based aerial images of the three study plots are available on Zenodo at https://zenodo.org/records/20811613, DOI: 10.5281/zenodo.20811613.

## Appendix A

**TABLE A1:** Changes in crown areas for 50 sampled trees in the Kuiseb plots from 2020 to 2025.

	Kuiseb 1 crown area			Kuiseb 2 crown area		
	2020 (sqm)	2025 (sqm)	Change (sqm)	2020 (sqm)	2025 (sqm)	Change (sqm)
	8.9	25.8	17.0	74.0	71.4	−2.6
	14.8	18.1	3.3	37.5	57.2	19.6
	236.8	250.9	14.1	135.7	193.1	57.4
	33.3	38.3	5.0	27.2	62.9	35.7
	138.1	139.9	1.7	19.0	28.8	9.7
	231.9	246.2	14.3	14.0	23.1	9.1
	57.3	126.8	69.5	128.3	149.8	21.4
	74.3	120.8	46.6	95.0	109.6	14.6
	180.4	170.4	−10.0	39.8	39.7	0.0
	22.0	24.8	2.8	45.4	127.1	81.7
	57.1	79.2	22.1	29.8	51.5	21.8
	54.1	83.0	28.9	138.4	225.2	86.9
	129.5	154.1	24.6	24.5	100.1	75.6
	24.4	31.9	7.5	76.4	156.6	80.1
	87.2	71.2	−16.0	307.4	335.9	28.5
	49.1	50.4	1.2	60.7	70.3	9.5
	99.1	96.1	−2.9	2.1	16.7	14.6
	17.9	20.0	2.1	9.1	24.3	15.2
	149.4	210.1	60.7	2.8	16.2	13.4
	8.1	29.3	21.2	605.7	626.4	20.8
	151.6	197.4	45.8	6.3	17.9	11.6
	15.5	23.4	7.8	112.2	115.6	3.5
	18.6	28.6	10.0	392.6	446.6	54.0
	447.5	512.2	64.7	68.6	130.4	61.8
	216.7	270.5	53.8	9.7	21.8	12.1
	165.2	199.9	34.7	121.3	165.6	44.4
	7.9	8.9	1.0	79.9	82.9	3.0
	12.5	20.3	7.8	44.6	89.6	45.1
	11.6	19.6	8.0	11.6	27.4	15.8
	49.9	70.6	20.6	34.4	63.3	28.9
	30.7	38.9	8.2	193.4	187.5	−5.9
	31.4	38.2	6.9	126.6	140.5	13.9
	193.4	214.8	21.4	76.4	91.9	15.5
	155.4	221.8	66.4	20.3	29.6	9.3
	56.2	97.1	41.0	12.5	16.7	4.2
	124.4	126.6	2.2	26.3	36.3	10.0
	102.2	107.7	5.5	14.4	23.6	9.2
	18.3	15.3	−2.9	66.9	156.2	89.4
	22.5	31.3	8.8	34.7	87.2	52.5
	9.0	15.1	6.1	31.0	127.5	96.6
	4.8	6.8	1.9	485.6	535.1	49.5
	47.3	63.3	16.0	173.1	185.5	12.4
	262.0	264.6	2.6	7.1	13.4	6.3
	27.2	61.8	34.6	24.5	29.5	5.0
	34.6	33.4	−1.2	10.5	23.3	12.8
	144.8	174.3	29.5	5.4	8.2	2.8
	129.4	142.2	12.9	133.2	133.9	0.8
	51.5	72.0	20.5	27.1	32.2	5.1
	76.6	80.3	3.7	18.8	21.6	2.8
	39.6	39.0	−0.7	150.3	219.9	69.6
**Sum**	**4331.6**	**5183.0**	**851.4**	**4391.9**	**5746.9**	**1355.0**
